# The research progress of the synergistic effect of Epstein-Barr virus and *Helicobacter pylori* infection in the occurrence and development of gastric cancer

**DOI:** 10.3389/fonc.2026.1807636

**Published:** 2026-07-31

**Authors:** Liming Gan, Liping Gong, Shan Wei, Junshan Deng

**Affiliations:** Department of Gastrointestinal and Burn Surgery, The First Affiliated Hospital of Hunan Traditional Chinese Medical College (Hunan Provincial Directly Affiliated Hospital of Traditional Chinese Medicine), Zhuzhou, Hunan, China

**Keywords:** Epstein-Barr virus, gastric cancer, *Helicobacter pylori*, research progress, synergistic effect

## Abstract

Gastric cancer (GC) is a malignant tumor characterized by high morbidity and mortality rates worldwide. The development of GC is a complex process influenced by various factors. Helicobacter pylori (HP) and Epstein-Barr virus (EBV) are recognized as pivotal contributors to this malignancy. HP instigates chronic inflammation, disrupts the gastric mucosal barrier, and triggers epigenetic modifications and aberrant cell signaling pathways, thereby facilitating GC progression. Conversely, EBV infection is specifically associated with Epstein-Barr virus-associated gastric cancer (EBVaGC), a distinct subtype of GC. EBV contributes to tumorigenesis by expressing latent membrane proteins, EBV nuclear antigens, and non-coding RNA, resulting in the hypermethylation of tumor suppressor genes in host cells and the rewiring of cellular metabolism. Historically, HP and EBV infections were considered distinct risk factors for gastric cancer. Nevertheless, recent research indicates a synergistic relationship between these pathogens in gastric cancer development, wherein they collaboratively influence key signaling pathways, bolster gastric cancer stem cell characteristics, and remodel the tumor microenvironment to expedite tumor advancement. The inflammatory milieu induced by HP infection fosters EBV colonization and reactivation. Consequently, comprehending the combined mechanism of gastric cancer initiation by EBV and HP infections holds paramount importance for gastric cancer treatment and prevention. This review comprehensively examines the synergistic interplay of HP and EBV in gastric cancer onset and progression, consolidates recent advancements in relevant research models and therapeutic approaches, and outlines prospective research avenues in this domain.

## 1 Introduction

Gastric cancer ranks among the leading causes of cancer-related mortality globally, with particularly elevated incidence rates in East Asia, including China, Japan, and South Korea. This phenomenon is closely associated with regional dietary practices, environmental influences, and a high prevalence of HP infection. HP is a Gram-negative bacterium capable of colonizing the acidic milieu of the stomach, with over 40% of the global population infected by this pathogen (1). Through its key virulence factors, including cytotoxin-related gene A (*cagA*) and vacuolating toxin A (VacA), HP activates various oncogenic signaling pathways, such as JAK-STAT, NF-κB, Wnt/β-catenin, and MAPK. This activation disrupts the normal processes of proliferation, apoptosis, and DNA repair in gastric epithelial cells, thereby playing a central role in the progression from chronic gastritis to atrophic gastritis, intestinal metaplasia, dysplasia, and ultimately gastric cancer (1). Additionally, HP lipopolysaccharide upregulates *MDM2* through the TLR4/MAPK/AP-1 pathway and promotes gastric cancer progression (2).

In addition to bacterial infections, viruses are implicated in the development of gastric cancer. The EBV,a human herpesvirus, has been recognized as the causative agent of EBVaGC, which accounts for approximately 10% of gastric cancer cases globally (3). EBVaGC represents an independent molecular subtype characterized by a complex interplay among its immune infiltration pattern, PD-L1 regulatory mechanisms, and epigenetic landscape, thereby establishing a distinct virus-driven tumorigenic logic. In terms of the immune microenvironment, EBVaGC displays a “hot tumor” phenotype, marked by robust CD8^+^ T cell infiltration and a notable presence of tertiary lymphoid structures, comprising 39.7% of the tumor microenvironment (4). However, this is paradoxically accompanied by terminal T cell depletion and elevated expression of immune checkpoint molecules, resulting in a scenario described as “immune hot but functionally suppressed.” PD-L1 exhibits abnormally high expression levels in this subtype; the TCGA data indicate that EBVaGC features local amplification of the 9p24.1 chromosomal region, which harbors the *CD274* (PD-L1) and *PDCD1LG2* (PD-L2) genes (5). Furthermore, approximately 85% of EBV-positive tumors test positive for PD-L1, a proportion significantly higher than that observed in other subtypes. At the epigenetic level, EBVaGC is characterized by a CpG island methylation phenotype (CIMP-H). Infection with EBV induces extensive hypermethylation of promoter CpG islands due to aberrant DNA methyltransferase activity, resulting in the high-frequency silencing of tumor suppressor genes such as *p16*, *FHIT*, and *WWOX*. Consequently, patients with CIMP-H exhibit significantly poorer prognoses (6). EBV disrupts host cell signal transduction by expressing latent membrane proteins (LMPs), Epstein-Barr virus nuclear antigens (EBNAs), and non-coding RNAs, including EBERs and miRNAs. This disruption induces hypermethylation of tumor suppressor gene promoters, leading to their silencing, and allows the virus to evade host immune surveillance, ultimately contributing to gastric cancer development (7, 8). Notably, Virus-induced metabolic reprogramming plays a crucial role in the malignant transformation of cells. Amino acids serve as a source of nitrogen and carbon for biosynthesis, while also fulfilling the energy demands of tumor cells that undergo rapid growth. The metabolic alterations induced by EBV virus infection frequently resemble those observed in cancer cells, including glutamine-dependent, aspartate-dependent, arginine-assisted, and active serine/proline metabolism. These changes facilitate both rapid viral replication and the accelerated proliferation of tumor cells (9).

Infection by HP and EBV, as distinct pathogens, can induce substantial alterations in host cell signaling pathways and cause DNA damage. Historically, these infections have been viewed as independent risk factors for gastric cancer (5, 10). However, accumulating evidence indicates the existence of co-infection, which may collaboratively influence the malignant phenotype of gastric cancer by modulating the tumor microenvironment (TME) and impacting the host’s response to immunotherapy, including immune checkpoint inhibitors (ICIs) (1, 11, 12). Co-infection with HP and EBV can induce significant structural alterations in gastric epithelial cells, leading to the upregulation of *TFF1*, *VIL1*, and *Lgr5*. This process promotes cell proliferation and tissue morphogenesis while enhancing the susceptibility of gastric epithelial cells to EBV infection (13). Concurrently, the two pathogens synergistically increase the expression of the autophagy-related protein LC3B and modify the activities of critical signaling pathways, including AKT/mTOR. These interconnected effects may facilitate tumor proliferation and contribute to increased treatment resistance (7, 11, 12, 14).

HP significantly alters the structure of gastric flora through its virulence factors, diminishes microbial diversity, and establishes a pro-inflammatory microenvironment, which drives chronic gastric mucosal lesions toward malignant transformation (15). EBV infection perpetuates immune responses, elevates pro-inflammatory cytokines such as IL-1β and TNF-α, compromises the gastric epithelial barrier, and fosters a niche favorable for tumor growth (16). Notably, co-infection with HP and EBV can synergistically enhance inflammatory signaling pathways, collectively generating a tumor-promoting microenvironment. Additionally, EBV infection exacerbates the substantial reduction of bacterial species in the stomach and further disrupts the ecological balance of the bacterial flora (17, 18). Each factor contributes to gastric microbial imbalance, and through the synergistic interaction of inflammatory signaling pathways, they collaboratively facilitate the progression of the gastric microenvironment from chronic inflammation to gastric cancer.

As our understanding of the carcinogenic mechanisms associated with HP and EBV deepens, and as gastric cancer molecular classification—such as the inclusion of the EBV-positive subtype in the TCGA classification—advances, intervention strategies targeting these pathogens, along with precision immunotherapy based on pathogen status, have emerged as a new frontier in gastric cancer research (1, 3). Furthermore, the development of innovative research models, including patient-derived gastric organoids, and the advancement of nanotherapeutic technologies, such as antimicrobial peptide-functionalized nanozymes, offer promising avenues to elucidate the synergistic mechanisms of HP and EBV while facilitating the creation of novel treatment strategies (13, 19). This article thoroughly examines the mechanisms by which EBV and HP infections contribute to the onset and progression of gastric cancer. It systematically reviews the current state of research and offers a theoretical foundation for personalized treatment strategies for patients with dual infections.

## 2 The synergistic carcinogenic mechanism of EBV and HP

### 2.1 Interactions of molecular pathways

**Figure 1:**
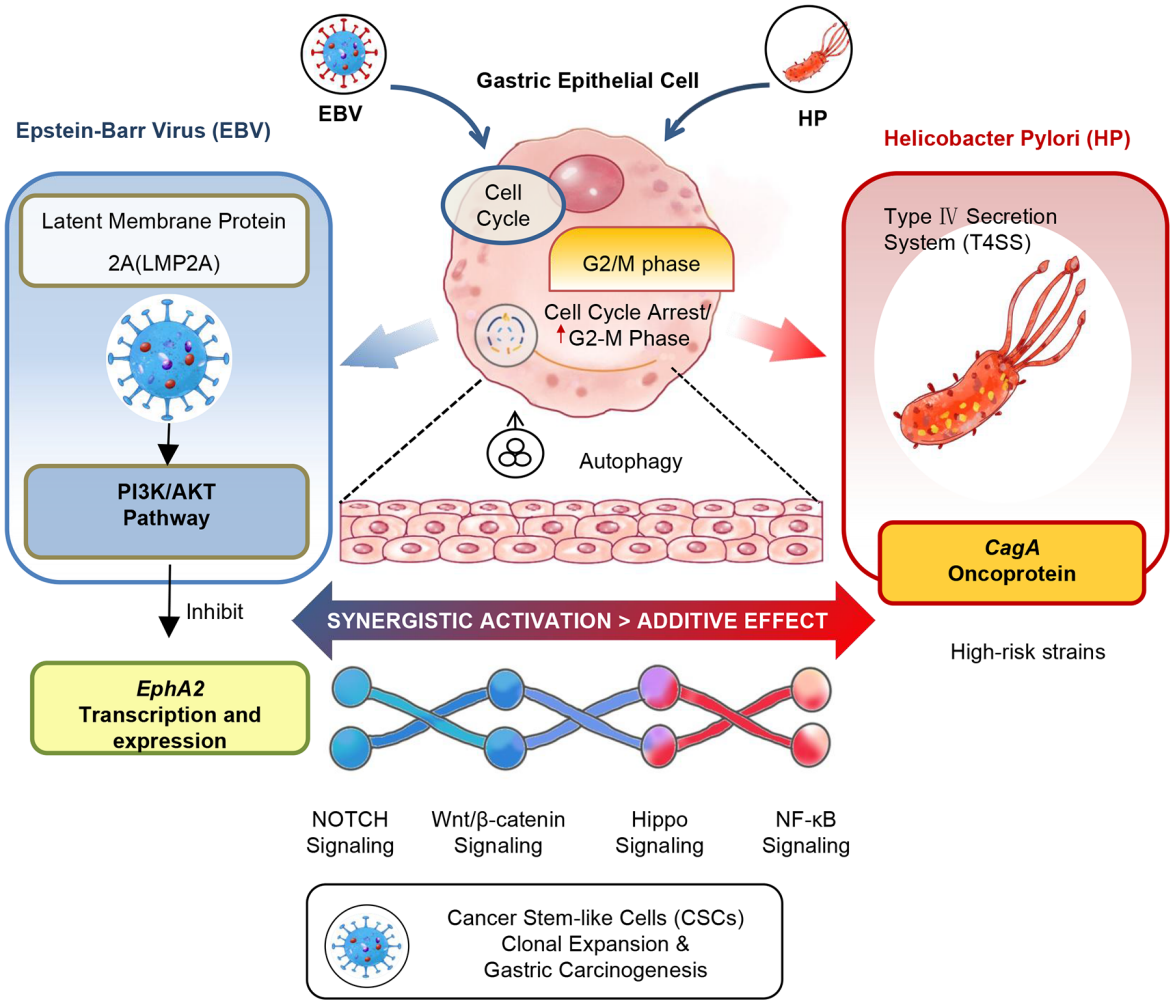
The synergistic mechanism of molecular pathways in the development of gastric cancer resulting from EBV and HP co-infection.

Co-infection with EBV and HP can synergistically modulate multiple critical signaling pathways, thereby facilitating the development of gastric cancer (13, 20) (**Figure 1**). Research indicates that these two pathogens collectively influence pathways associated with stem cell maintenance, including Notch, Wnt/β-catenin, Hippo, and NF-κB. This interaction promotes the proliferation of cancer cell populations exhibiting stem cell characteristics, significantly increasing cancer incidence (21). Notably, in the context of co-infection, the aberrant activation of these pathways demonstrates a pronounced synergistic effect, rather than a mere additive effect (14, 22) (**Table 1**).

**Table 1:** Synergistic mechanism of EBV and HP in gastric cancer development.

Mechanism of action	The primary contribution of EB virus	The primary contribution of *Helicobacter pylori*	Synergy	References
Signal Pathway Regulation	LMP2A inhibits EphA2 phosphorylation through the PI3K-AKT pathway.	*CagA* protein induces entry into host cells, disrupting multiple signaling pathways.	Co-regulate the Notch, Wnt, Hippo, and NF-κB pathways to promote gastric cancer stem cell properties.	(13, 14, 21–24)
Immune Microenviron-ment Remodeling	Induction of PD-L1 expression; miR-BART20-3p targets PPARα to upregulate IL-6	Induce chronic inflammation and recruit immunosuppre-ssive cells	Collaboratively create an immunosuppressive microenvironment to enhance immune evasion.	(17, 45–48, 72)
Epigenetic modifications	Induces genome-wide hypermethylation of CPG islands, silencing tumor suppressor genes	Inflammation induces the accumulation of abnormal methylation	Co-create an epigenetic landscape conducive to malignant transformation	(7, 59–62)
Cell Cycle Regulation	Regulate cell cycle progression, influencing the balance between proliferation and apoptosis	Specific strains cause cell cycle arrest	Co-infection alters cell cycle distribution and promotes abnormal proliferation.	(22, 28, 37–40)

In the context of EBV infection, the latent membrane protein 2A (LMP2A) encoded by the virus activates the PI3K-AKT signaling pathway. This activation inhibits the transcription of the *EphA2* gene and promotes its autophagic degradation, resulting in a significant downregulation of *EphA2* protein levels. The diminished expression of *EphA2* impairs its capacity to drive tumor cell proliferation, migration, and invasion, thereby inhibiting the progression of gastric cancer and cell division. Concurrently, the reduced phosphorylation of *EphA2* at Ser901 obstructs the activation of the JNK (c-Jun N-terminal kinase) pathway, which is crucial for inducing EBV lysis and reactivation. Consequently, LMP2A not only restricts the malignant phenotype of tumors through the downregulation of *EphA2* but also prevents EBV from entering lytic replication, thereby maintaining latent viral infection (5, 23, 24). This finding elucidates the underlying mechanism for the relatively favorable prognosis associated with EBV-related gastric cancer and offers a potential therapeutic target for lysis induction therapy in this context.

EBV latency is categorized into three distinct gene expression programs. Latent type I exclusively expresses non-coding RNAs, including *EBNA1* and EBERs. Latent type II builds upon type I by increasing the expression of LMP2A, BARTs, and other genes. Latent type III, which is primarily observed in post-transplantation lymphoproliferative diseases, expresses all latent genes (25, 26). Concurrently, EBVaGC is categorized as either latent type I or II, expressing *EBNA1*, EBERs, BART miRNAs, and LMP2A (25). The carcinogenic mechanism of EBVaGC involves viral latent genes, such as LMP2A, which activate the STAT3/NF-κB pathway, leading to abnormal hypermethylation of the host genome and the silencing of tumor suppressor genes, including *PTEN*. Additionally, genetic abnormalities, such as *PIK3CA* mutations and *JAK2*/PD-L1 amplification, collectively contribute to tumor initiation and progression (25, 27, 28).

HP infection relies heavily on its virulence factors, particularly the *CagA* protein, to trigger the carcinogenic process. Variations in toxicity among various clinical strains have a substantial impact on their carcinogenic capacity (29, 30). Research indicates that clinical isolates of HP from regions with a high incidence of gastric cancer predominantly consist of virulent strains, which exhibit distinct molecular characteristics of virulence factors (31–33). Through its cytotoxin-associated gene pathogenicity island (cagPAI) and type IV secretion system (T4SS), HP directly disrupts the normal functions of host cells, thereby inducing inflammatory responses and promoting cell transformation (34–36).

Co-infection with EBV and HP can influence the proliferation and survival of gastric epithelial cells by modulating the autophagy pathway and regulating cell cycle progression. Specifically, co-infection with certain strains of HP and EBV accelerates the transition of gastric epithelial cells from the G1 phase to the G2/M phase. This phenomenon primarily results from the synergistic effects of HP and EBV. The CagA protein, encoded by the *cagA* gene of HP, activates multiple signaling pathways, including PI3K/AKT, downregulates negative cell cycle regulators such as p27Kip1, and promotes the expression of cyclin D1. These actions collectively facilitate the progression of cells from the G1 restriction point into the S phase (28). Additionally, EBV’s latent membrane protein 2A collaborates with *MYC* to further downregulate p27Kip1, thereby enhancing the G1/S phase transition and enabling a greater number of cells to enter the S phase and successfully navigate the G2/M checkpoint (37). Co-infection also significantly down-regulated the expression of pro-apoptotic genes, including *APAF-1* and *BAX*, while inhibiting the accumulation of apoptotic cells in the sub-G1 phase. This dual effect, characterized by “accelerating cell cycle progression” and “inhibiting apoptotic clearance,” results in an increased proportion of cells in the G2/M phase. Concurrently, the relative decrease of cells in both the G1 and sub-G1 phases influences tumor occurrence and development by stimulating cell division and proliferation (13, 38–40).

When EBV and HP co-infect gastric epithelial cells, timely implementation of standardized treatment is crucial. The current first-line empirical treatment regimen consists of anti-HP eradication therapy, which includes a proton pump inhibitor, bismuth, and two antibiotics. Regular monitoring through gastroscopy is essential; if lesions progress to EBV-positive gastric cancer, EBV status testing is recommended. Radical surgery should be performed based on tumor stage, and comprehensive treatment should incorporate immune checkpoint inhibitors, such as PD-1 monoclonal antibodies, along with SOX chemotherapy regimens. Failure to administer timely treatment may allow the two pathogens to persist, leading to the abnormal activation of multiple key signaling pathways and resulting in significant synergistic effects. Furthermore, this activation accelerates the cell division cycle, thereby promoting the occurrence and progression of gastric cancer.

### 2.2 Tumor microenvironment and immune regulation

**Figure 2:**
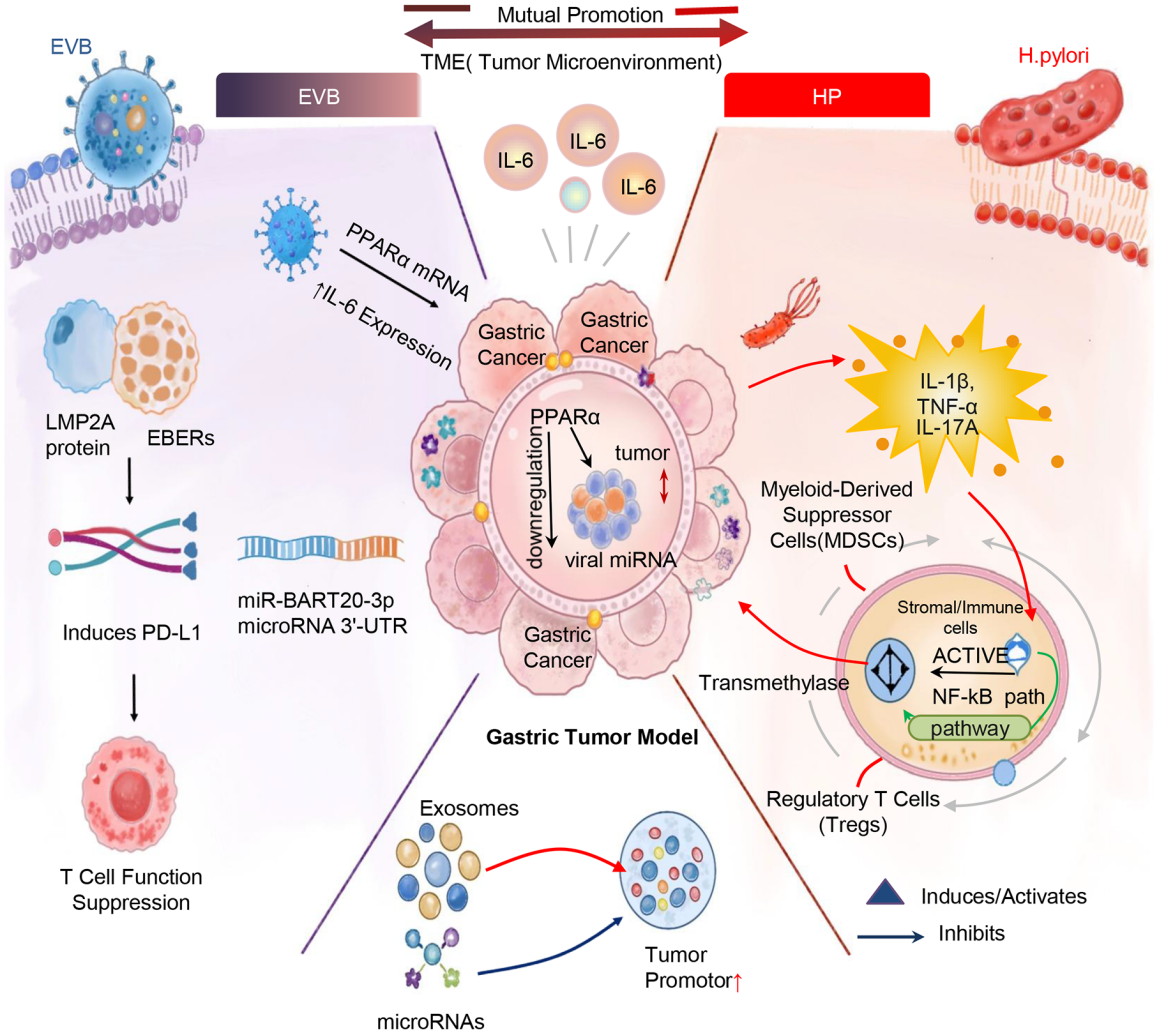
Infection with EBV and HP induces alterations in the gastric tumor cell microenvironment and facilitates immune evasion.

Co-infection with EBV and HP synergistically establishes an immunosuppressive tumor microenvironment that facilitates immune evasion and the malignant progression of tumor cells (41) (**Figure 2**). Single-cell analysis revealed that the virome characteristics in gastric cancer tissues are closely associated with alterations in immune cell composition. Notably, the abundance of acutely transformed retroviruses in HP-positive samples increased significantly, indicating a potential for synergistic carcinogenesis involving both bacteria and viruses (42, 43).

EBV infection facilitates immune evasion through various mechanisms. The LMP2A protein, along with the EBERs it encodes, can induce elevated expression of PD-L1 in both tumor cells and infiltrating immune cells, thereby inhibiting T cell function (12). Furthermore, EBV-encoded miR-BART20-3p suppresses PPARα expression by targeting the 3’-UTR of PPARα mRNA, which leads to an increase in IL-6 levels and contributes to the formation of an immunosuppressive microenvironment (44). PPARα is a ligand-activated nuclear receptor transcription factor that primarily exerts anti-inflammatory and immunoregulatory effects in gastric epithelial cells; it negatively regulates the expression of pro-inflammatory factors such as IL-6 by competitively binding to NF-κB coactivators or directly interfering with NF-κB transcriptional activity. When miR-BART20-3p down-regulates PPARα, its inhibitory effect on NF-κB is alleviated, resulting in a marked increase in IL-6 transcription. Elevated levels of IL-6 contribute to immune suppression at multiple levels by activating the STAT3 signaling pathway within the tumor microenvironment. Specifically, STAT3 directly enhances the expression of immune checkpoint molecules such as PD-L1 and CTLA-4, which inhibits the activation and cytotoxic functions of CD8^+^ T cells (45–48). Additionally, IL-6/STAT3 signaling facilitates the differentiation and recruitment of myeloid-derived suppressor cells (MDSCs) and regulatory T cells (Tregs), while also inducing the polarization of tumor-associated macrophages (TAMs) toward the immunosuppressive M2 phenotype. Collectively, these effects synergistically diminish the anti-tumor immune response and create a microenvironment that promotes tumor immune escape (49–51).

HP infection primarily facilitates the development of the tumor microenvironment by inducing chronic inflammation. Upon infecting the gastric mucosa, HP activates various inflammatory pathways, resulting in the substantial release of pro-inflammatory cytokines and chemokines, including IL-1β, IL-17A, and TNF-α. These inflammatory mediators stimulate DNA methyltransferase through the activation of the nuclear factor kappa B (NF-κB) pathway and an increase in nitric oxide levels, thereby promoting the methylation of tumor suppressor gene promoters (52, 53).

During co-infection, chronic inflammation induced by HP fosters the accumulation of reactive oxygen species and DNA double-strand breaks. Concurrently, EBV impairs the DNA damage response repair pathway and diminishes the efficiency of cell cycle checkpoints. Together, these factors synergistically enhance genomic instability (17, 54). Gankyrin serves as a critical bridge molecule in the context of HP infection, which can enhance Gankyrin expression. This upregulation promotes cell proliferation and metastasis by interacting with tumor suppressor proteins, such as p53 and Rb, thereby facilitating their degradation. Notably, Gankyrin knockdown in HP-infected cells significantly diminished their *in vivo* tumorigenicity, while downregulation of Gankyrin resulted in increased levels of NF-κB, pRb, and p53. These findings suggest that Gankyrin plays a pivotal role in HP-mediated gastric cancer progression by modulating both inflammatory and tumor suppressive pathways. Additionally, HP can synergize with Epstein-Barr virus (EBV)-induced epigenetic modifications via the Wnt/β-catenin pathway, further disrupting the regulation of cell polarity and barrier homeostasis (22, 55, 56). Multiple mechanisms intertwine to create a synergistic malignant platform characterized by the “acceleration of inflammation, apparent silencing, and accumulation of DNA damage,” collectively hastening the evolution of gastric cancer.

EBV infection modifies the tumor microenvironment and suppresses T lymphocyte activity. In contrast, HP infection triggers the release of numerous inflammatory factors. The co-infection of these two pathogens significantly intensifies the immunosuppressive characteristics of the tumor microenvironment, thereby exacerbating immune evasion and promoting the malignant progression of gastric cancer cells.

### 2.3 Alterations in epigenetic modifications

**Figure 3:**
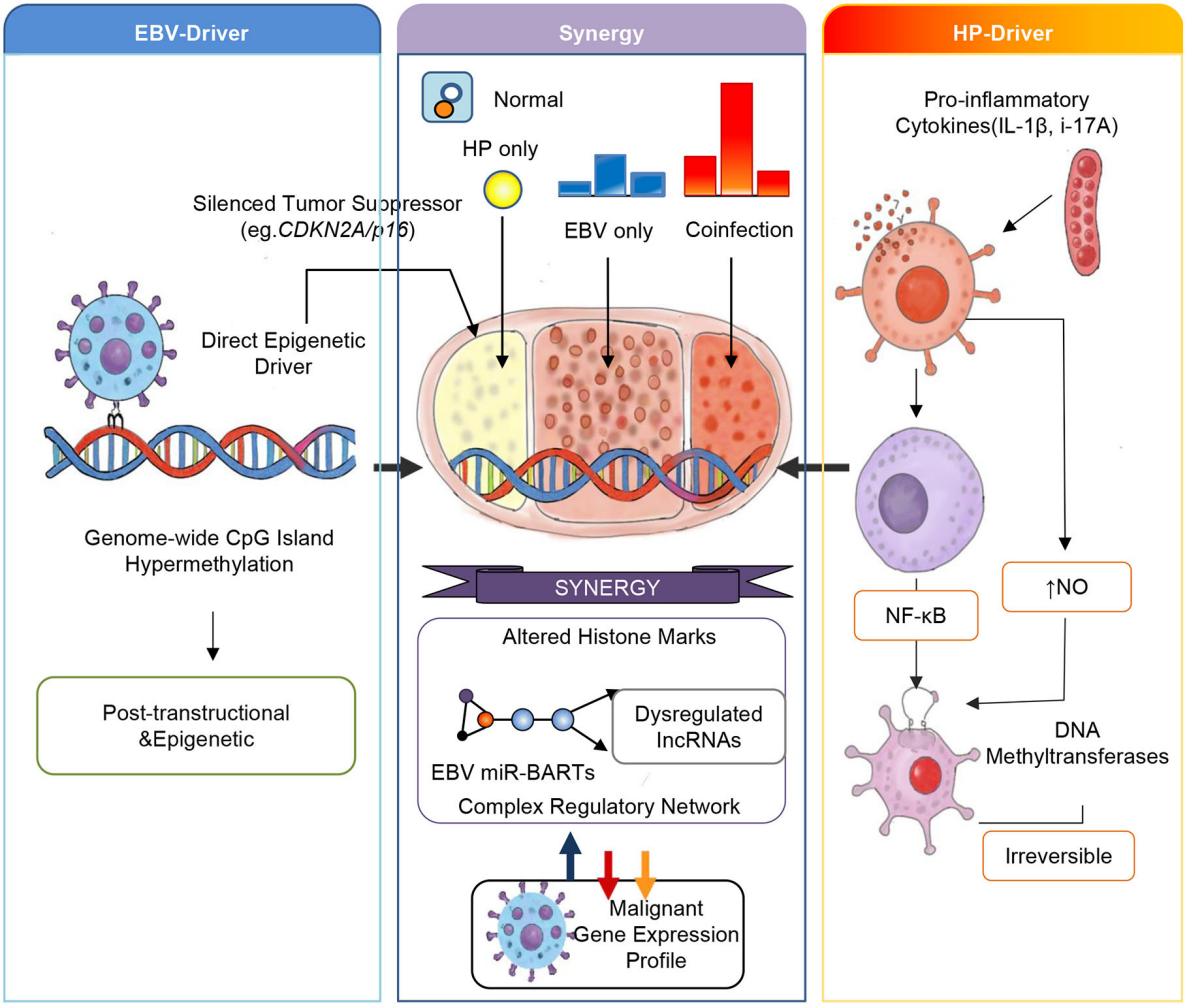
Infection with EBV and HP induces alterations in the epigenetic modifications of gastric epithelial cells.

Epigenetic alterations, particularly DNA methylation, represent a fundamental mechanism through which EBV and HP contribute to gastric carcinogenesis (57–59) (**Figure 3**). In EBVaGC, a prominent characteristic is the genome-wide hypermethylation of CpG islands, which is believed to result from epigenetic reprogramming directly induced by the virus. This process leads to the silencing of numerous tumor suppressor genes, including *CDKN2A/p16 (*60–62).

HP infection primarily induces an accumulation of abnormal methylation through the promotion of chronic inflammation. Such inflammatory states can disrupt epigenetic regulatory mechanisms. Prolonged inflammatory signals, including IL-1β and TNF-α, may result in the hypermethylation of tumor suppressor gene promoter regions, either through the accumulation of reactive oxygen species(ROS) or by directly inhibiting the activity of DNA methyltransferases(DNMTs). This process contributes to genome instability and carcinogenesis (63, 64). Notably, even after the eradication of HP, certain methylation marks may remain irreversible, underscoring the critical importance of early intervention in the prevention of gastric cancer (65).

When co-infected with EBV and HP, the two pathogens synergistically establish an epigenetic environment in the gastric mucosa that favors malignant transformation. Whole-genome methylation analysis reveals that the gastric mucosal tissue of co-infected patients exhibits more extensive and profound methylation alterations. This epigenetic reprogramming directly influences the expression of critical tumor-related genes, thereby further advancing the carcinogenic process (66–68).

In addition to DNA methylation, histone modifications and noncoding RNAs significantly contribute to the synergistic carcinogenesis associated with these two pathogens. Research indicates that HP infection can induce the expression of multiple long non-coding RNAs (lncRNAs) in host cells, thereby altering the host’s gene expression profile. EBV encodes various viral microRNAs, such as miR-BARTs, which directly participate in the post-transcriptional regulation of host gene expression. Collectively, these alterations in epigenetic mechanisms form a complex regulatory network that facilitates the onset and progression of gastric cancer (11, 69–71).

Further elucidation of the synergistic interaction between EBV and HP infection in the onset and progression of gastric cancer is warranted.

Signal Pathway Regulation: Co-infection with HP and EBV synergistically enhances the acquisition of gastric cancer stem cell (GCSC) characteristics by modulating critical signaling pathways, including Notch, Hippo, NF-κB, and Wnt. HP promotes the formation of GCSCs through the activation of the Wnt/β-catenin pathway and simultaneously stimulates the NF-κB signaling pathway, leading to the upregulation of *ONECUT2*, which further enhances GCSC characteristics. Conversely, EBV influences the maintenance and differentiation of GCSCs by regulating the Notch and Hippo pathways. Together, these pathogens coordinate the regulation of stemness-related signaling networks within the inflammatory microenvironment, driving the self-renewal and maintenance of GCSCs while accelerating the progression of gastric cancer (14).

Immune Microenvironment Remodeling: HP and EBV co-infection collaboratively establishes an immunosuppressive microenvironment through dual mechanisms. HP employs *CagA* and other virulence factors to perpetually activate the NF-κB and STAT3 signaling pathways, resulting in the upregulation of immunosuppressive molecules, including regulatory T cells, myeloid-derived suppressor cells, and IL-10. Concurrently, EBV activates the NF-κB and JAK/STAT pathways via latent membrane proteins, leading to elevated expression of PD-L1. Together, these interactions synergistically amplify inflammatory signals, enhance immune evasion, and promote the progression of gastric cancer (17, 72).

Epigenetic modifications: Co-infection with HP and EBV synergistically enhances DNA methyltransferase activity, resulting in hypermethylation of the promoter regions of tumor suppressor genes, thereby silencing their expression. Concurrently, hypomethylation of specific repetitive sequences induces genome instability, collectively establishing an epigenetic environment that facilitates malignant transformation and promotes tumor invasion behavior (7, 59).

Cell Cycle Regulation: Co-infection with HP and EBV leads to the upregulation of the oncoprotein Gankyrin, which in turn increases the levels of cyclin D1 (CCND1) and proliferating cell nuclear antigen (PCNA). Concurrently, this co-infection downregulates tumor suppressors such as p53 and pRB, while inhibiting the expression of the pro-apoptotic genes *APAF1* and *BAX*. As a result, more cells progress through the G1/S checkpoint and accumulate in the G2/M phase, while apoptosis is suppressed. This process ultimately results in a characteristic distribution marked by an increased proportion of cells in the G2/M phase and a decreased proportion in the sub-G1 phase, thereby driving the malignant proliferation of gastric epithelial cells (22, 28).

## 3 Research models and technological advances

### 3.1 Applications of gastric organoid models

Previous research predominantly utilized animal models and conventional two-dimensional cell culture methods, both of which possess significant limitations. Animal models, primarily involving mice and gerbils, exhibit species differences that hinder their ability to accurately replicate the complex processes associated with human diseases. For instance, EBV, a herpes virus that exclusively infects human epithelial cells, cannot effectively infect most non-human hosts, thereby severely restricting the development of relevant animal models. Furthermore, traditional two-dimensional cell culture fails to authentically replicate the three-dimensional structure and functionality of gastric tissue, as it lacks the intricate interactions between cells and the extracellular matrix (73, 74). These constraints have impeded advancements in understanding the mechanisms underlying co-infection by HP and EBV.

In recent years, the advancement of gastric organoid models has emerged as a significant tool for investigating co-infection by EBV and HP (**Table 2**). A research team from Fudan University successfully developed patient-derived gastric organoid models, which include normal gastric organoids (NGO) and tumor gastric organoids (TGO), both of which closely mimic the structure and function of gastric tissue. By employing high-precision microinjection technology to introduce EBV and HP into these organoids, the researchers established an effective *in vitro* co-infection model. This model offers novel tools and methodologies for exploring the mechanisms underlying the co-infection of HP and EBV in gastric cancer (13).

**Table 2:** Current advances in EBV and HP synergy research field.

Research methods and models	Application advantages	Key findings in the EBV-HP study	Technical limitations and challenges	References
Gastric organoid model	Maintain three-dimensional structure and highly simulate gastric tissue function	Coinfection promotes HP internalization and EBV lytic replication; synergistically enhances cell proliferation.	Long cultivation cycle; individual variations may affect result consistency.	(13, 73, 74)
CRISPR-Cas9 screening	Unbiased identification of host factors	*DSC2* was identified as the primary receptor for EBV infection of epithelial cells.	Off-target effects may occur, requiring substantial verification efforts.	(81–84)
Multi-omics integrated analysis	Systemic Analysis of Viral-Host Interactions	Establish a diagnostic model for viral biomarkers; Identify a positive correlation between HERV-K and gastric cancer progression.	Low-abundance virus detection lacks sufficient sensitivity, and data analysis is complex.	(87–92)
Microinjection Technology	Precise control of infection timing and dosage	Achieve targeted infection within organoids to study the spatial distribution of pathogens	High technical barriers and significant operational challenges	(13)

More importantly, co-infection facilitated the internalization of HP. In single infections, HP was predominantly distributed on the luminal surface of the organoid. However, during co-infection, the localization of HP shifted from the surface to the interior of the organoid, where it co-localized with EBV-infected cells. This observation indicates that EBV infection may augment the invasive capacity of HP into gastric epithelial cells (13, 75–77).

Moreover, the gastric organoid model elucidated the effects of co-infection on the EBV life cycle. The study revealed that HP co-infection markedly increased the expression of the EBV latent antigen EBNA1and the lytic activator BZLF1. This suggests that HP co-infection not only promotes the latent infection of EBV but also triggers viral lytic replication. These findings hold considerable importance for elucidating the dynamic changes of EBV in gastric carcinogenesis (57, 78–80).

### 3.2 Gene editing and receptor identification

Gene editing technology, particularly the CRISPR-Cas9 system, has achieved significant advancements in identifying host factors associated with EBV infection (**Table 2**). The study revealed that genome-wide CRISPR-Cas9 screening successfully identified *Desmocollin 2 (DSC2)* as the primary receptor for EBV entry into epithelial cells. This discovery addresses a longstanding scientific question regarding the mechanism by which EBV effectively infects epithelial cells. The knockdown of *DSC2* significantly diminished EBV infection in nasopharyngeal and gastric epithelial cell lines. Conversely, restoration of *DSC2* expression resulted in either the re-emergence or substantial enhancement of infection. Furthermore, *DSC2* directly interacts with the EBV glycoprotein H/glycoprotein L via its extracellular domain, particularly the pre-EC-EC2 region, which is amenable to targeting by polyclonal antibodies, thereby inhibiting EBV infection in primary epithelial cells. These findings not only enhance the understanding of EBV entry into epithelial cells but also lay the groundwork for the development of targeted therapies and the construction of animal models (81–84).

In the realm of HP research, gene editing technology has been extensively employed to investigate the interactions between host factors and HP infection. Utilizing gene knockout and transgenic expression techniques, researchers have identified several critical host genes that play roles in HP attachment, internalization, and inflammatory responses. These investigations offer significant insights into the pathogenesis of HP and inform the development of targeted treatment strategies (85, 86).

### 3.3 Multi-omics and bioinformatics analysis

The advancement of high-throughput sequencing technology and bioinformatics analysis methods has enabled researchers to investigate the roles of EBV and HP in gastric carcinogenesis at a systemic level. By integrating relevant data, Chinese scholars have systematically mapped the virome distribution associated with gastric cancer through transcriptome sequencing and single-cell analysis. They identified key pathogenic viruses, including human endogenous retrovirus (HERV-K), EBV, and cytomegalovirus (HCMV), for the first time. A correlation exists between toxicity and gastric cancer, with specific HERV subfamilies, such as LTR5_Hs_1q22 and HERVS71_19q13.22, exhibiting positive expression in gastric cancer. These subfamilies are significantly associated with invasive gastric cancer and can promote cell proliferation *in vitro* (87, 88). The 30 viral markers screened, including HERV-K and HERV-W, effectively distinguish gastric cancer from adjacent tissue, with HERV-K being significantly enriched in diffuse gastric cancer. This discovery offers new perspectives for the early diagnosis of gastric cancer (89–92) (**Table 2**).

Additionally, bioinformatics analysis revealed intricate interactions between the virus and the host genome. Visual analysis using IGV identified that the genome sequence of HERV-K exhibits high homology with several human genes, including *ABCC2*, *SGCD*, and *PRODH*. Notably, the HERV-K homologous fragment located in the PRODH promoter region may influence gene expression through its binding to transcription factors such as *SOX2.* These findings uncover novel mechanisms by which viral integration impacts host gene expression (74, 87, 93).

## 4 Clinical significance and therapeutic prospects

### 4.1 Pathogen-targeted therapeutic strategies

A comprehensive understanding of the synergistic carcinogenic mechanisms of EBV and HP offers opportunities for the development of novel targeted treatment strategies. Regarding HP-targeted therapy, research indicates that standard triple therapy, new quadruple therapy, and 10-day sequential therapy can effectively eliminate virulent HP strains, with the 10-day sequential therapy being particularly advantageous. Extensive clinical implementation has demonstrated that HP eradication significantly reduces the incidence of gastric cancer in specific populations (94, 95).

Researchers are investigating various targeting strategies for EBV infection. Studies indicate that sodium butyrate (NaB) treatment can enhance EBV activation in EBV-positive gastric cancer cells by stimulating the MAPK signaling pathway while inhibiting the NF-κB pathway. This results in a notable upregulation of key latency and lytic phase gene expressions. Mechanistically, research has revealed that the core transcription factor gene *EGR1* plays a role in viral activation by facilitating the transcription of EBV lytic genes *BZLF1* and *BRLF1*. These findings provide an experimental foundation for the development of “lytic induction therapy” (78, 96, 97).

Simultaneously, treatment strategies based on epigenetics demonstrate considerable potential. The primary epigenetic driving force behind EBVaGC is the virus-induced CIMP-H. In this context, the EBV-encoded LMP2A enhances *DN*MT1 activity via phosphorylation of STAT3, leading to hypermethylation of over 3,000 promoter CpG islands. This process silences critical tumor suppressor genes, including *p16*, *PTEN*, and *PTPN6* (98, 99). Consequently, epigenetic therapeutic strategies employ two classes of drugs: the DNA methyltransferase inhibitors (DNMTi) decitabine and azacitidine, which can reverse the hypermethylation of tumor suppressor gene promoters and restore their functional expression (100). Research has shown that decitabine treatment can selectively revive the transcriptional activity of retinoic acid receptors (RARα/RARβ) in EBVaGC, leading to increased tumor cell apoptosis through activation of the retinoic acid pathway. Simultaneously, demethylation can disrupt the hypermethylation status of the EBV genome, triggering the reactivation of the lytic cycle and exposing latent viruses to tumor antigens (98, 101). Histone deacetylase inhibitors (HDACi) like vorinostat alter chromatin structure by enhancing histone acetylation, facilitating the re-expression of tumor suppressor genes, and orchestrating multi-faceted transcriptional regulation (102). As preclinical investigations accumulate, the combined use of DNMTi and HDACi has shown promise in synergistically reprogramming epigenetically silenced genes, paving the way for a novel approach to the precise treatment of EBVaGC.

### 4.2 Immunotherapy strategies

Gastric cancer associated with co-infection by EBV and HP demonstrates distinct advantages in immunotherapy. A comprehensive global multi-center retrospective analysis revealed that EBVaGC exhibits heightened sensitivity to immune checkpoint inhibitor (ICI) treatment, potentially due to its high PD-L1 expression rate and extensive lymphocyte infiltration. In a case involving a patient with EBVaGC who received first-line chemotherapy combined with ICI, an objective response rate (ORR) of 49% was achieved, alongside median progression-free survival (PFS) and overall survival (OS) of 8.3 months and 38 months, respectively. Furthermore, the expression level of PD-L1 was closely linked to ICI efficacy. Multivariate analysis confirmed that a PD-L1 combined positive score (CPS) of ≥5 is significantly associated with improved survival outcomes, with a notable difference in median PFS observed between patients with PD-L1 CPS ≥5 and those with PD-L1 CPS <5 (103). A single-center study conducted by Korean researchers in 2025 confirmed that EBV positivity serves as an independent predictor of survival benefit for patients undergoing first-line nivolumab in combination with chemotherapy. This efficacy advantage is closely associated with elevated PD-L1 CPS expression in patients (104). Additionally, a retrospective study published in 2025, which involved 77 HER2-negative EBV-positive patients, demonstrated that the median PFS for the immunotherapy combined with chemotherapy group (9.28 months) was significantly longer than that of the chemotherapy-only group (6.07 months) (105). In summary, clinical research data indicate that EBV-positive gastric cancer represents a distinct molecular subtype characterized by a heightened sensitivity to immunotherapy, primarily due to its immune-inflammatory microenvironment marked by elevated PD-L1 expression. Although the anticipated efficacy has not been realized in studies involving small sample sizes or specific agents, such as camrelizumab administered alone, existing evidence suggests that PD-L1 expression levels remain a crucial biological marker for predicting the potential benefits of first-line immune combination chemotherapy or ICI treatment alone in patients with EBV-positive gastric cancer (12, 68, 106–108). These findings provide a robust evidence-based foundation for the clinical development of precision immunotherapy strategies.

In addition to conventional immune checkpoint inhibitors, researchers are investigating alternative immunotherapy strategies (109–111). For instance, adoptive T cell therapy that targets EBV-specific antigens is currently being assessed in clinical trials. This approach facilitates the targeted destruction of EBV-positive tumor cells by isolating and expanding T cells capable of recognizing viral antigens, such as EBNA1 and LMP2, before reinfusing them into patients. Likewise, therapeutic vaccines aimed at EBV latency antigens are under development, with the goal of preventing or treating EBV-related malignancies.

### 4.3 Precise diagnosis and prognosis assessment

In the context of accurate diagnosis, EBER *in situ* hybridization (EBER-ISH) is regarded as the gold standard for identifying EBVaGC. However, this detection method is expensive and necessitates stringent laboratory techniques, which somewhat restricts its clinical applicability. In recent years, Next-generation sequencing (NGS) technology has emerged as a valuable tool for diagnosing EBVaGC. Research indicates that the results from NGS and EBER-ISH are highly consistent, exhibiting a positive coincidence rate of 98% and a negative coincidence rate of 99.9%. This evidence suggests that NGS can reliably identify EBVaGC, thereby offering a more efficient and cost-effective alternative for clinical detection (112, 113).

In biomarker research, alongside traditional viral markers such as EBERs and EBNA1, an increasing number of studies have begun to examine the prognostic and predictive significance of PD-L1 expression, tumor mutation burden (TMB), and viral non-coding RNA in EBVaGC. Notably, PD-L1 in conjunction with CPS has demonstrated considerable prognostic stratification value in EBVaGC (114–116).

PD-L1 expression is currently the most widely utilized companion diagnostic biomarker in the field of immunotherapy for gastric cancer (**Table 3**). The CPS from immunohistochemistry (IHC) testing is routinely employed in clinical practice to identify populations that may benefit from ICI (117). Although HP 16S rRNA is extensively used as a research tool for the molecular diagnosis of HP infection and the analysis of gastric microbiota characteristics in gastric cancer patients, it has not yet been established as an independent clinical testing item within standard diagnostic pathways. Its clinical translation remains in the exploratory phase. Non-invasive gastric cancer screening utilizing stool and plasma ctDNA methylation markers is currently undergoing prospective validation (NCT06979895, NCT06943768). The detection of methylation has amassed significant evidence supporting its utility in prognosis prediction and minimal residual disease (MRD) monitoring. Studies have confirmed the prognostic value of ctDNA methylation markers such as *SPG20*, *FBN1*, and *SDC2*. Furthermore, population simulations have demonstrated that the SPOGIT model can decrease the diagnosis rate of advanced gastrointestinal cancer by 92% (118). The unique CIMP-H methylation phenotype observed in EBVaGC suggests that the demethylating agent decitabine has specific therapeutic potential for restoring tumor suppressor gene expression and activating the retinoic acid signaling pathway (101). EBV-encoded miRNAs, including miR-BART19-3p, miR-BART5-5p, and miR-BART20-3p, are crucial for tumor proliferation, migration, and cell cycle regulation, and they have progressed to functional verification and biomarker development (50, 70). Furthermore, the combination of circulating small RNAs, such as hsa-miR-3916 and hsa-miR-181d-5p, with PD-L1 CPS can elevate the AUC for predicting responses to gastric cancer immunotherapy to 0.89, thereby offering a novel multi-dimensional strategy for the precise screening of beneficial patient groups (119).

**Table 3:** Summary of gastric cancer biomarkers based on clinical trials.

Category	Biomarker	Sample source	Experimental stage/key data	Main uses	References
Classic	PD-L1 CPS	Tumor tissue(IHC)	Phase III clinical trials have been approved(CheckMate-649,KEYNOTE series, etc.)	Prediction of immunotherapy efficacy/companion diagnosis	(120–125)
Classic	CEA,CA19-9,CA72-4	Serum	Routine clinical application	Auxiliary diagnosis/therapeutic effect monitoring	(44, 126, 127)
Potential	ctDNA methylation (*SPG20,FBN1*,*SDC2*,*TFPI2*,*SEPT9*)	Plasma	Prognostic verification cohort (sensitivity and specificity await large-scale verification)	Prognosis prediction/MRD monitoring	(101, 125, 126)
Potential	Polygenic methylation index	Plasma	Phase II observational (NCT06979895)	Treatment response monitoring/adaptive treatment orientation	(101, 120–125)
Potential	Fecal methylation combined with Hp susceptibility genes	Feces	Multicenter observational property(NCT06943768, n=9,654)	Early screening for gastric cancer	(98, 120, 126, 128)
Potential	*RIMS1* DNA methylation	Tissue/Feces	Multicenter prospective high-risk population validation	Risk prediction of Gastric cancer	(98, 129, 130)
Potential	hsa-miR-3916,hsa-miR-181d-5p	Plasma	Queue validation (n=91,AUC=0.82-0.89)	Prediction of immunotherapy response	(98, 124, 131)
Potential	EBV-miR-BART19-3p	Tumor tissue	*In vitro*/*in vivo* functional verification	EBVaGC proliferation promotion/therapeutic target	(118, 125, 127)
Potential	EBV-miR-BART5-5p (Targeting *RORA*)	Tumor tissue	*In vitro* functional verification	EBVaGC diagnostic/therapeutic targets	(118, 125)
Potential	EBV-miR-BART20-3p(targeting PPARα)	Tumor tissue	*In vitro* mechanism verification	EBVaGC proliferation and migration promotion	(101, 132, 133)
Potential	BAF index (Microbiota ratio based on bacterial 16S rRNA)	Serum exosomes	Case-control (AUC = 0.760)	Early diagnosis/prognosis immune microenvironment assessment	(121–124, 128)
Potential	*ORAOV1* (regulating PD-L1 through *MYC*)	Tumor tissue	*In vitro*/retrospective cohort	Promote immune escape/potential therapeutic targets	(117, 129)

## 5 Conclusions and prospects

EBVaGC constitutes approximately 10% of gastric cancer cases globally, with notable regional and population variations in this percentage (134, 135). Co-infection with EBV and HP can elevate the risk of gastric cancer by approximately 2.57 times, indicating potential additive or synergistic carcinogenic effects (80). Geographically, the reported prevalence of EBVaGC is notably high in East Asia, particularly in Japan and South Korea, as well as in Latin America, while it remains significantly lower in certain areas, such as Iran. This disparity suggests the influence of environmental or host genetic factors that are not yet fully understood (135, 136). At the population level, the incidence of EBVaGC in men is approximately double that in women. This malignancy predominantly arises in the proximal stomach, specifically the cardia and gastric body. Pathologically, it primarily presents as the diffuse type and is more prevalent among younger individuals, as well as among African and Hispanic populations (137, 138). These epidemiological characteristics indicate that EBVaGC represents a condition with extensive global distribution, synergistic infection dynamics, and considerable clinical heterogeneity. Therefore, effective strategies for prevention, control, diagnosis, and treatment must be carefully tailored to account for regional and population-specific factors.

The study of co-infection by EBV and HP in relation to gastric cancer marks a shift from focusing on individual pathogens to exploring the intricate microbial networks involved in cancer. Current research not only elucidates the carcinogenic mechanisms of each pathogen but also highlights the synergistic effects between them. This “1 + 1>2” synergistic carcinogenic model offers new insights into the pathogenesis of gastric cancer (13, 139). Despite significant advancements, this field continues to face numerous challenges and future directions:

The specific molecular mechanisms underlying the synergistic effects of EBV and HP remain to be fully elucidated. Notably, the existence of a direct molecular interaction between these two pathogens, as well as their combined regulation of the epigenetic and signaling pathways in host cells, necessitates further investigation (26). Employing innovative organ model systems alongside real-time imaging technology could illuminate the dynamics of pathogen-pathogen-host interactions during co-infection.

Innovative treatment strategies for coinfected patients must be devised as current treatments primarily focus on a single pathogen. Research on combination treatment approaches for coinfected individuals is still nascent. Considering the synergistic mechanisms of carcinogenesis, the development of simultaneous targeted therapies for EBV and HP, or the integration of pathogen-targeted therapy with immunotherapy and epigenetic therapy, may be crucial in the future (39, 66, 109).

To enhance clinical translation, it is essential to establish more accurate risk stratification models. Current research indicates that co-infected patients may exhibit distinct clinicopathological characteristics and prognoses. However, the integration of co-infection status into clinical decision-making processes, as well as the development of individualized screening and treatment strategies for these patients, requires validation through large-scale prospective studies (38, 77).

Furthermore, prevention and early intervention strategies for EBV and HP co-infection merit further investigation. Given that HP eradication therapy has been shown to reduce the risk of gastric cancer, it is crucial to determine whether and how more proactive interventions are necessary for high-risk groups co-infected with EBV and HP. Addressing these significant clinical questions will be vital for future research (140).

Although substantial evidence supports a causal relationship between EBV and HP co-infection and the progression of gastric cancer, the prevailing theoretical framework predominantly relies on *in vitro* observational studies utilizing organoid models. This reliance presents limitations that cannot be overlooked in the strength of its causal argument. First, existing organoid systems struggle to accurately replicate the complex spatial architecture of the gastric mucosa, which encompasses epithelial-mesenchymal interactions, innervation, and vascular networks. Consequently, *in vitro* assessments of the complete biological effects of pathogenic microorganisms continue to encounter challenges related to insufficient modeling fidelity (13, 71).Secondly, organoid culture lacks a microenvironment that facilitates the dynamic interaction of natural immune components, such as macrophages, lymphocytes, and dendritic cells, along with the *in vivo* microbial community. This absence raises uncertainty regarding whether the synergistic inflammatory pathways identified *in vitro* are fully representative of *in vivo* conditions (95). Furthermore, the long-term coevolutionary relationship between EBV and HP imposes multidimensional selection pressures on the host’s genetic background and intragastric niche, which are challenging to replicate in static microinjection models (13, 141). Therefore, until conclusive functional validation in population cohorts and longitudinal causal evidence is achieved, caution is warranted in extrapolating the true causal relationship between EBV-HP co-infection and the progression of gastric cancer.

The ongoing advancement of precision medicine is poised to enhance personalized prevention, diagnosis, and treatment strategies that specifically target the unique subtypes of Epstein-Barr virus and Helicobacter pylori co-infection, thereby offering significant survival benefits to patients. Concurrently, comprehensive investigations into the synergistic mechanisms of EBV and HP will yield valuable insights into the role of complex microbial networks in the onset and progression of tumors. Ultimately, this research may lead to innovative approaches for the comprehensive prevention, control, and treatment of gastric cancer.

## Glossary

GC: Gastric cancer
HP: Helicobacter pylori
EBV: Epstein-Barr virus
EBVaGC: Epstein-Barr virus-associated gastric cancer
LMPs: latent membrane proteins
EBNAs: Epstein-Barr virus nuclear antigens
TME: Tumor microenvironment
LMP2A: Latent membrane protein 2A
EphA2: Ephrin type-A receptor 2
T4SS: type IV secretion system
MDSCs: myeloid-derived suppressor cells
DNMTs: DNA methyltransferases
lncRNAs: long non-coding RNAs
GCSC: gastric cancer stem cell
EBNA1: Epstein-Barr Nuclear Antigen 1
BZLF1: BamHI Z fragment left reading frame 1
BRLF1: BamHI R fragment left reading frame 1
LMPs: latent membrane proteins
EBNAs: Epstein-Barr virus nuclear antigens
HERV-K: human endogenous retrovirus
DNMTi: DNA methyltransferase inhibitors
HDACi: Histone deacetylase inhibitors
PFS: progression-free survival
OS: overall survival
CPS: combined positive score
EBER-ISH: EBER *in situ* hybridization
NGS: next-generation sequencing
TMB: tumor mutation burden
IHC: immunohistochemistry
ICI: immune checkpoint inhibitor
MRD: minimal residual disease
MDM2: Mouse Double Minute 2
EphA2: Ephrin type-A receptor 2
EBERs: EBV-encoded RNAs
BARTs: BamHI A Rightward Transcripts
cagA: cytotoxin-related gene A
APAF-1: Apoptotic protease activating factor-1
BAX: Bcl-2-associated X protein
PD-L1: Programmed Death-Ligand 1
CTLA-4: Cytotoxic T-Lymphocyte-Associated protein 4
HERV-W: Human Endogenous Retrovirus group W
HERV-K: Human Endogenous Retrovirus group K
HCMV: cytomegalovirus
EGR1: Early Growth Response 1
DNMT1: DNA Methyltransferase 1
p16/CDKN2A: Cyclin-Dependent Kinase Inhibitor 2A
PTEN: Phosphatase and Tensin Homolog
PTPN6: Protein Tyrosine Phosphatase Non-Receptor Type 6
MRD: minimal residual disease
SPG20: Spastic Paraplegia 20
FBN1: Fibrillin 1
SDC2: Syndecan 2
TFPI2: Tissue Factor Pathway Inhibitor 2
SEPT9: Septin 9
RIMS1: Regulating Synaptic Membrane Exocytosis 1
CIMP-H: CpG Island Methylator Phenotype-High
SPOGIT: Screening for the Presence of Gastrointestinal Tumors
CCND1: cyclin D1
PCNA: proliferating cell nuclear antigen
NGO: normal gastric organoids
TGO: tumor gastric organoids
ONECUT2: One Cut Homeobox 2
PPARα: Peroxisome Proliferator-Activated Receptor Alpha
DSC2: Desmocollin 2
FHIT: Fragile Histidine Triad
WWOX: WW Domain Containing Oxidoreductase
CD274: CD274 molecule
PDCD1LG2: PDCD1 ligand 2
TFF1: Trefoil Factor 1
VIL1: Villin 1
Lgr5: Leucine-Rich Repeat-Containing G-Protein Coupled Receptor 5
TCGA: The Cancer Genome Atlas
CTLA4: Cytotoxic T-Lymphocyte-Associated Protein 4
SOX2: SRY (sex-determining region Y)-box 2
MAPK: Mitogen-Activated Protein Kinases
ABCC2: ATP-binding cassette subfamily C member 2
SGCD: delta-sarcoglycan
PRODH: proline dehydrogenase (oxidase) 1
HDACi: Histone deacetylase inhibitors
NGS: Next-generation sequencing
MYC: Myelocytomatosis oncogene
ORAOV1: Oral Cancer Overexpressed 1
VacA: vacuolating toxin A
PIK3CA: 5-Bisphosphate 3-Kinase Catalytic Subunit Alpha Phosphatidylinositol-4
JAK2: Janus Kinase 2
RORA: RAR-related orphan receptor alpha
cagPAI: cytotoxin-associated gene pathogenicity island
AUC: Area Under the Curve
MRD: Measurable Residual Disease
